# *EHP* Paper of the Year, 2013

**DOI:** 10.1289/ehp.1307850

**Published:** 2013-12-01

**Authors:** Hugh A. Tilson

**Affiliations:** Editor-in-Chief, EHP, E-mail: tilsonha@niehs.nih.gov

*Environmental Health Perspectives* (*EHP*) established the Paper of the Year Award in 2008 to recognize high-quality papers published in the journal (Tilson 2008). Starting in 2011, *EHP* decided to recognize two papers each year (Tilson 2011). The *EHP* Classic Paper of the Year is the research article, commentary, or review that is the most highly cited over the preceding 60 months. The winner of the 2013 *EHP* Classic Paper of the Year, announced in September (Tilson 2013), was “Effects of Particulate Matter on Genomic DNA Methylation Content and *iNOS* Promoter Methylation” by Letizia Tarantini, Matteo Bonzini, Pietro Apostoli, Valeria Pegoraro, Valentina Bollati, Barbara Marinelli, Laura Cantone, Giovanna Rizzo, Lifang Hou, Joel Schwartz, Pier Alberto Bertazzi, and Andrea Baccarelli (Tarantini et al. 2009).

The second paper to be recognized each year is the *EHP* Paper of the Year. This award recognizes the most highly cited paper published during the preceding year. *EHP* is pleased to announce that the 2013 Paper of the Year is “450K Epigenome-Wide Scan Identifies Differential DNA Methylation in Newborns Related to Maternal Smoking during Pregnancy” by Bonnie R. Joubert, Siri E. Håberg, Roy M. Nilsen, Xuting Wang, Stein E. Vollset, Susan K. Murphy, Zhiqing Huang, Cathrine Hoyo, Øivind Midttun, Lea A. Cupul-Uicab, Per M. Ueland, Michael C. Wu, Wenche Nystad, Douglas A. Bell, Shyamal D. Peddada, and Stephanie J. London (Joubert et al. 2012). This paper was published in the October 2012 issue of the journal.

It is well known that children whose mothers smoked during pregnancy are more likely to have health problems such as low birth weight and asthma. Joubert et al. addressed the hypothesis that the diverse effects of maternal smoking during pregnancy may involve epigenetic modifications such as DNA methylation. The investigators obtained cord blood samples from a pregnancy cohort in the Norwegian Mother and Child Study (MoBa), and they used the Illumina Methyl450K platform to examine whether methylation changes were associated with smoking during pregnancy. Joubert et al. found cord blood methylation of genes in a pathway important for the detoxification of chemicals in tobacco smoke. They also identified the methylation of novel genes not previously implicated in the response to tobacco smoke, one of which is involved in fundamental developmental processes. Work is now in progress to *a*) determine whether these changes persist in children at 7 years of age, *b*) examine the role of timing of exposure, and *c*) explore whether epigenetic inheritance plays a role in these findings. The authors are also coordinating an effort to bring together studies with data from the same platform for a meta-analysis of the effects of maternal smoking on newborns. The findings of Joubert et al. support the possibility that epigenetic mechanisms seen by DNA methylation may be associated with effects of maternal smoking during pregnancy.

*EHP* congratulates all the authors of this paper for their contribution to the environmental health science literature. This research is important because it demonstrates the value of using this approach to study mechanisms of epigenetic effects of *in utero* exposures to chemicals and other environmental factors.
